# Supplementary material for: Broadly neutralizing antibodies against emerging delta-coronaviruses
Source: bioRxiv. 2024 Apr 1:2024.03.27.586411. Preprint. [Version 2] doi: 10.1101/2024.03.27.586411 (PMC11014491; doi:10.1101/2024.03.27.586411)
Supplement: Supplement 1 [file NIHPP2024.03.27.586411v2-supplement-1.pdf]

bioRxiv preprint doi: https://doi.org/10.1101/2024.03.27.586411; this version posted April 1, 2024. The copyright holder for this preprint (which was not certified by peer review) is the author/funder, who has granted bioRxiv a license to display the preprint in perpetuity. It is made available under aCC-BY 4.0 International license.

Supplementary Figures

bioRxiv preprint doi: https://doi.org/10.1101/2024.03.27.586411; this version posted April 1, 2024. The copyright holder for this preprint (which was not certified by peer review) is the author/funder, who has granted bioRxiv a license to display the preprint in perpetuity. It is made available under aCC-BY 4.0 International license.

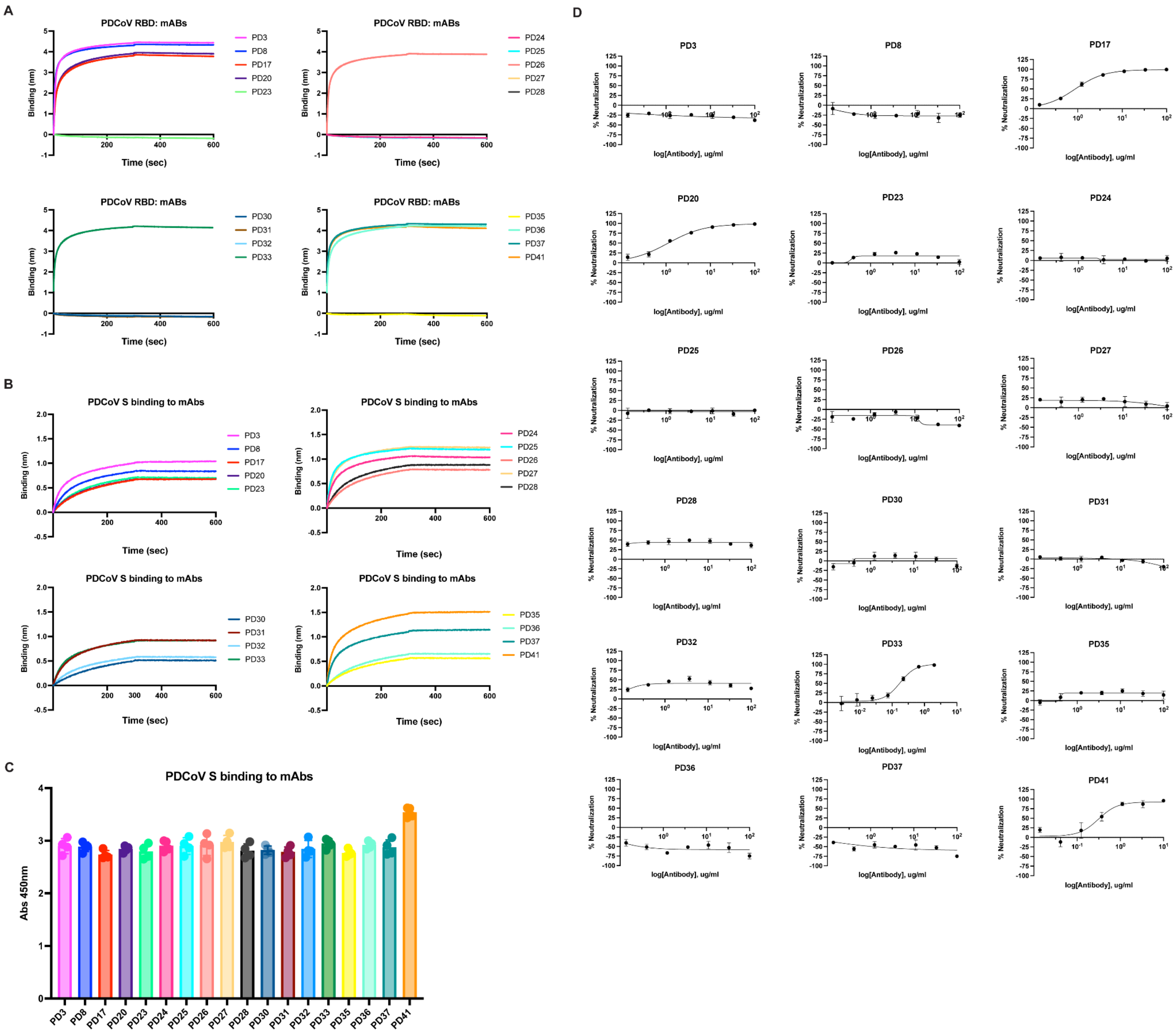

Figure S1. Screening for binding and neutralization of a panel of human mAbs (PD3, PD8, PD17, PD20, PD23, PD24, PD25, PD26, PD27, PD28, PD30, PD31, PD32, PD33, PD35, PD36, PD37, PD41) isolated from transgenic mice. A, Screening of isolated mAbs for binding to PDCoV RBD immobilized at the surface of BLI biosensors. **B,** Screening of isolated mAbs immobilized at the surface of BLI biosensors for binding to PDCoV S **C,** Evaluation of binding of the isolated mAbs to PDCoV S measured by ELISA. Each point represents the mean of technical quadruplets. Standard deviations shown as error bars. **D,** Dose-dependent mAb-mediated neutralization of PDCoV S VSV using HEK293T target cells transiently transfected with galline APN (gAPN). Each point represents the mean of technical triplicates. Standard deviations shown as error bars.

bioRxiv preprint doi: https://doi.org/10.1101/2024.03.27.586411; this version posted April 1, 2024. The copyright holder for this preprint (which was not certified by peer review) is the author/funder, who has granted bioRxiv a license to display the preprint in perpetuity. It is made available under aCC-BY 4.0 International license.

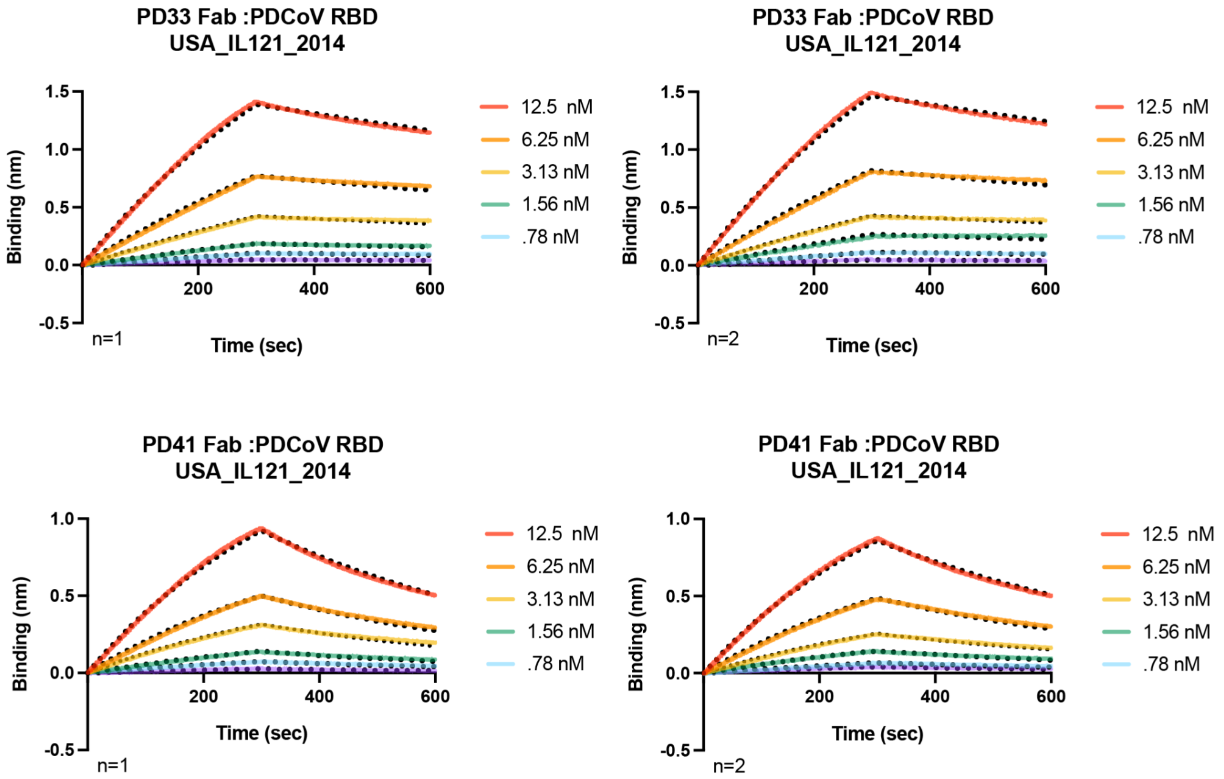

Figure S2: Kinetics of PD33 and PD41 Fabs binding to PDCoV RBD. BLI analysis of PD33 and PD41 Fab fragments binding to immobilized $PDCoV_{IL121\_2014}$ RBD immobilized onto Ni-NTA tips. Fits to the data are shown as black dotted lines and were used to determine the binding affinity (K_D) of the PD33 and PD41 Fab fragments to PDCoV RBD. Two biological replicates are shown for each Fab.

bioRxiv preprint doi: https://doi.org/10.1101/2024.03.27.586411; this version posted April 1, 2024. The copyright holder for this preprint (which was not certified by peer review) is the author/funder, who has granted bioRxiv a license to display the preprint in perpetuity. It is made available under aCC-BY 4.0 International license.

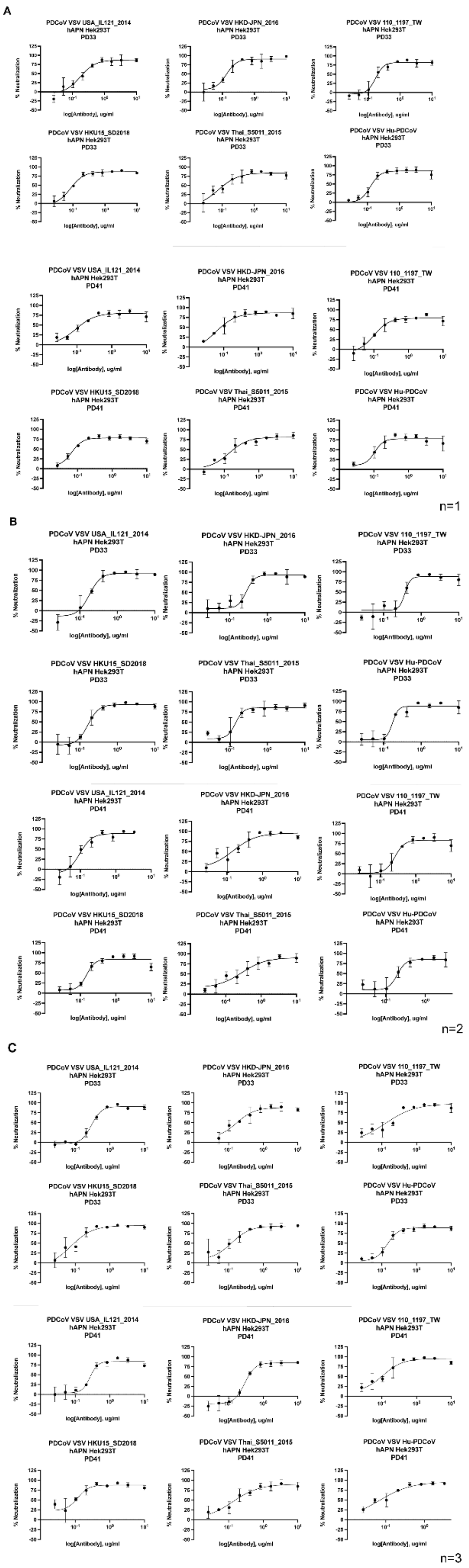

bioRxiv preprint doi: https://doi.org/10.1101/2024.03.27.586411; this version posted April 1, 2024. The copyright holder for this preprint (which was not certified by peer review) is the author/funder, who has granted bioRxiv a license to display the preprint in perpetuity. It is made available under aCC-BY 4.0 International license.

Figure S3. Assessment of PD33 and PD41 neutralization breadth. Dose-dependent mAb-mediated neutralization of PDCoV S VSV variants using HEK293T target cells transiently transfected with human APN. A-C are three independent runs with 3 different batches of pseudovirus.

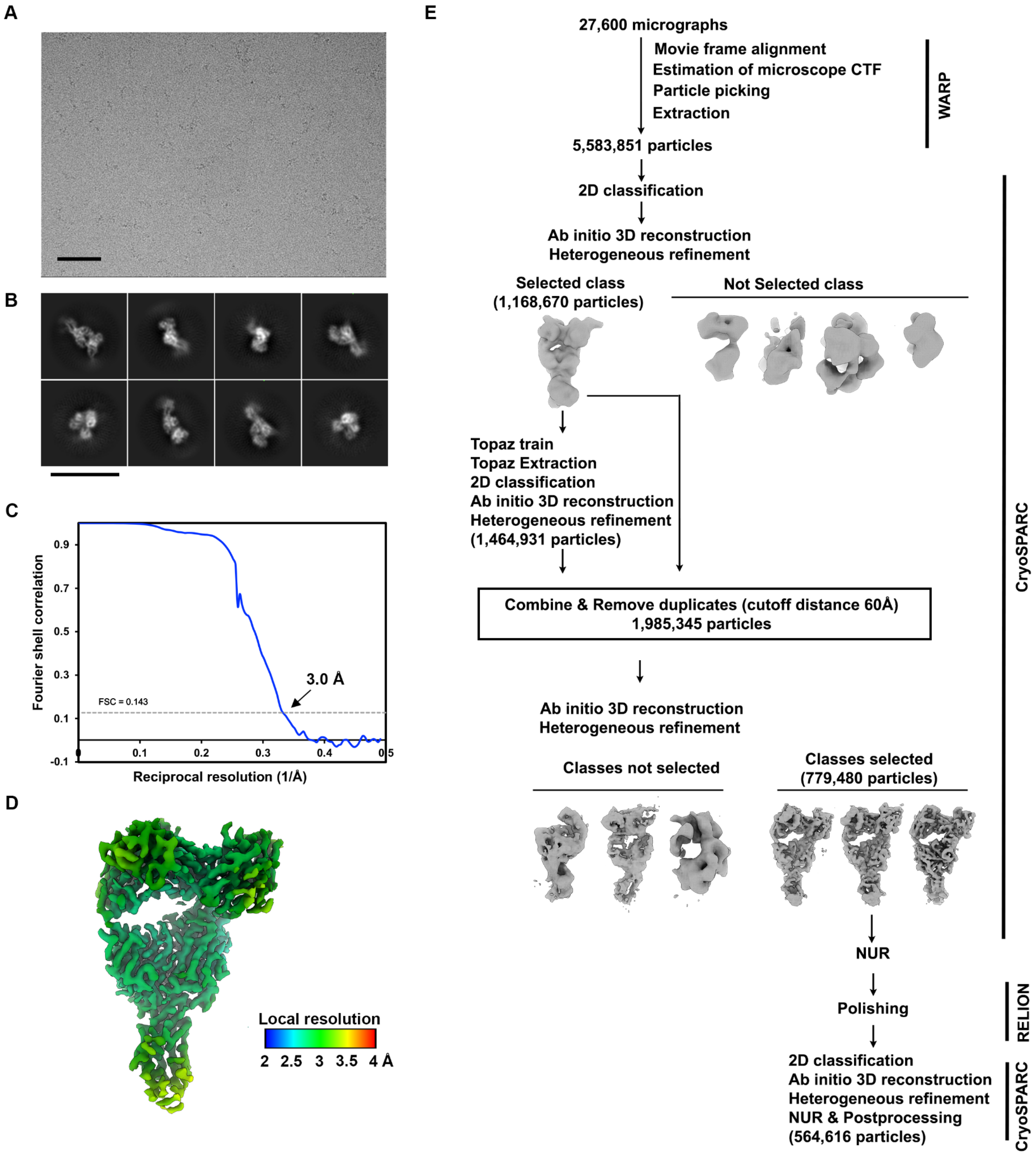

bioRxiv preprint doi: https://doi.org/10.1101/2024.03.27.586411; this version posted April 1, 2024. The copyright holder for this preprint (which was not certified by peer review) is the author/funder, who has granted bioRxiv a license to display the preprint in perpetuity. It is made available under aCC-BY 4.0 International license.

Figure S4: Cryo-EM data collection and refinement of PDCoV RBD$_{IL2014}$ bound to the PD33 Fab fragment. A, Representative electron micrograph (3.0 µm defocus, scale bar = 100 nm) **B,** 2D class averages (scale bar = 150 Å). **C,** Gold-standard Fourier shell correlation curve for the cryoEM reconstruction. The 0.143 cutoff is indicated with a gray dashed line. **D,** 3D reconstruction of PDCoV RBD bound to PD33 colored by local resolution calculated using CryoSPARC. **E,** Flow chart of the pipeline for processing. CTF: contrast transfer function; NUR: non-uniform refinement.

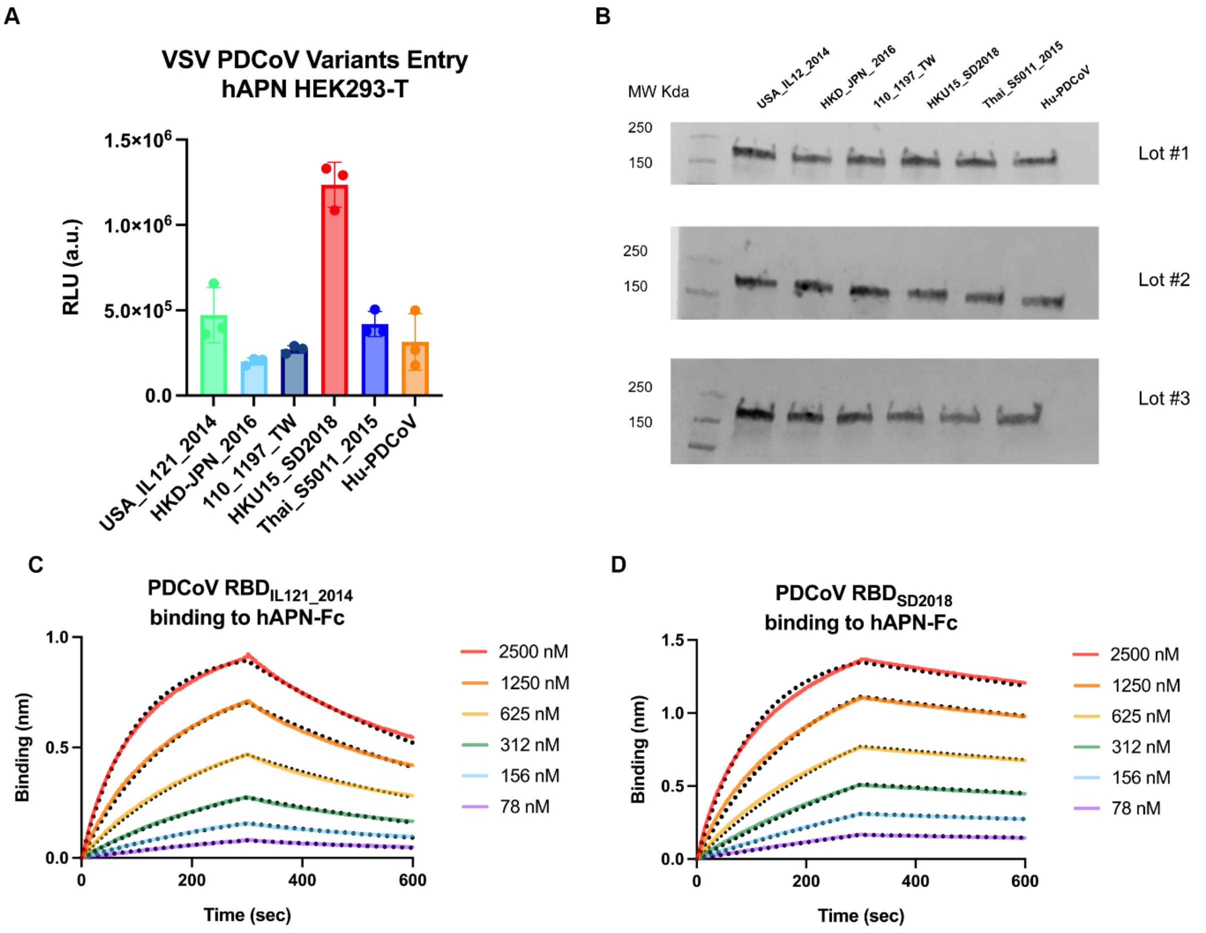

Figure S5. Functional characterization of PDCoV S variants. A, Entry of PDCoV S VSV variants into HEK293T target cells transiently transfected with human APN. **B,** Western blot quantification of PDCoV S incorporation in VSV pseudotypes for each of the three biological replicates used in panel A. **C-D,** BLI analysis of hAPN-Fc binding to the PDCoV$_{IL121_2014}$ RBD (left) or to the PDCoV$_{SD_2018}$ (right) immobilized onto Ni-NTA tips. Fits to the data are shown as black dotted lines and were used to determine the apparent binding affinity ($K_{D,app}$) of hAPN-Fc fragments to the two PDCoV RBDs.

bioRxiv preprint doi: https://doi.org/10.1101/2024.03.27.586411; this version posted April 1, 2024. The copyright holder for this preprint (which was not certified by peer review) is the author/funder, who has granted bioRxiv a license to display the preprint in perpetuity. It is made available under aCC-BY 4.0 International license.

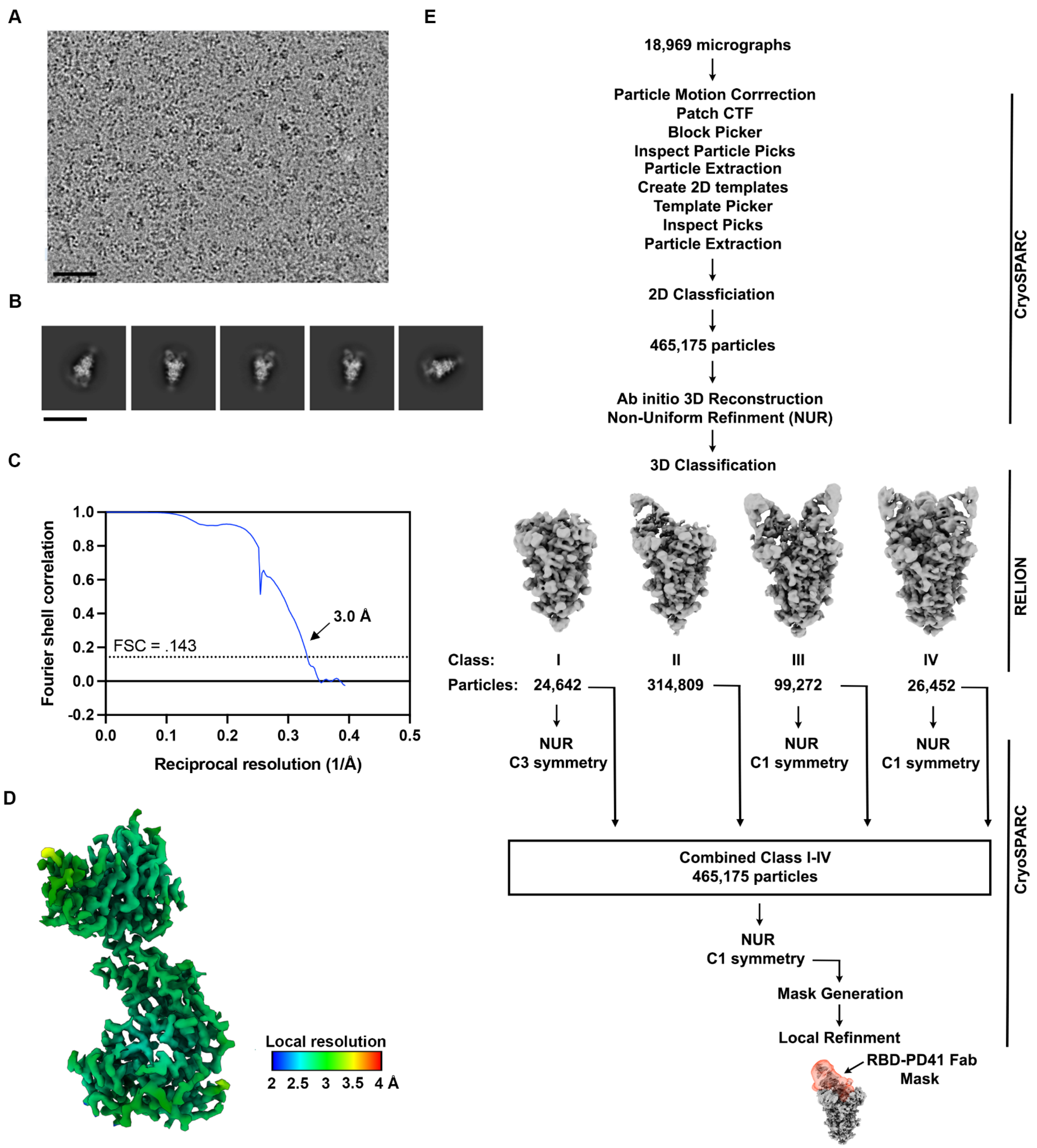

Figure S6: Cryo-EM data collection and refinement of PDCoV RBD$_{SD2018}$ bound to the PD41 Fab fragment. A, Representative electron micrograph (2.5 μm defocus, scale bar = 100 nm) **B,** 2D class averages (scale bar = 150 Å). **C,** Gold-standard Fourier shell correlation curve for the cryoEM reconstruction. The 0.143 cutoff is indicated with a black dashed line. **D,** 3D reconstruction of PDCoV RBD bound to PD41 colored by local resolution calculated using CryoSPARC. **E,** Flow chart of the pipeline for processing. CTF: contrast transfer function; NUR: non-uniform refinement.

bioRxiv preprint doi: https://doi.org/10.1101/2024.03.27.586411; this version posted April 1, 2024. The copyright holder for this preprint (which was not certified by peer review) is the author/funder, who has granted bioRxiv a license to display the preprint in perpetuity. It is made available under aCC-BY 4.0 International license.

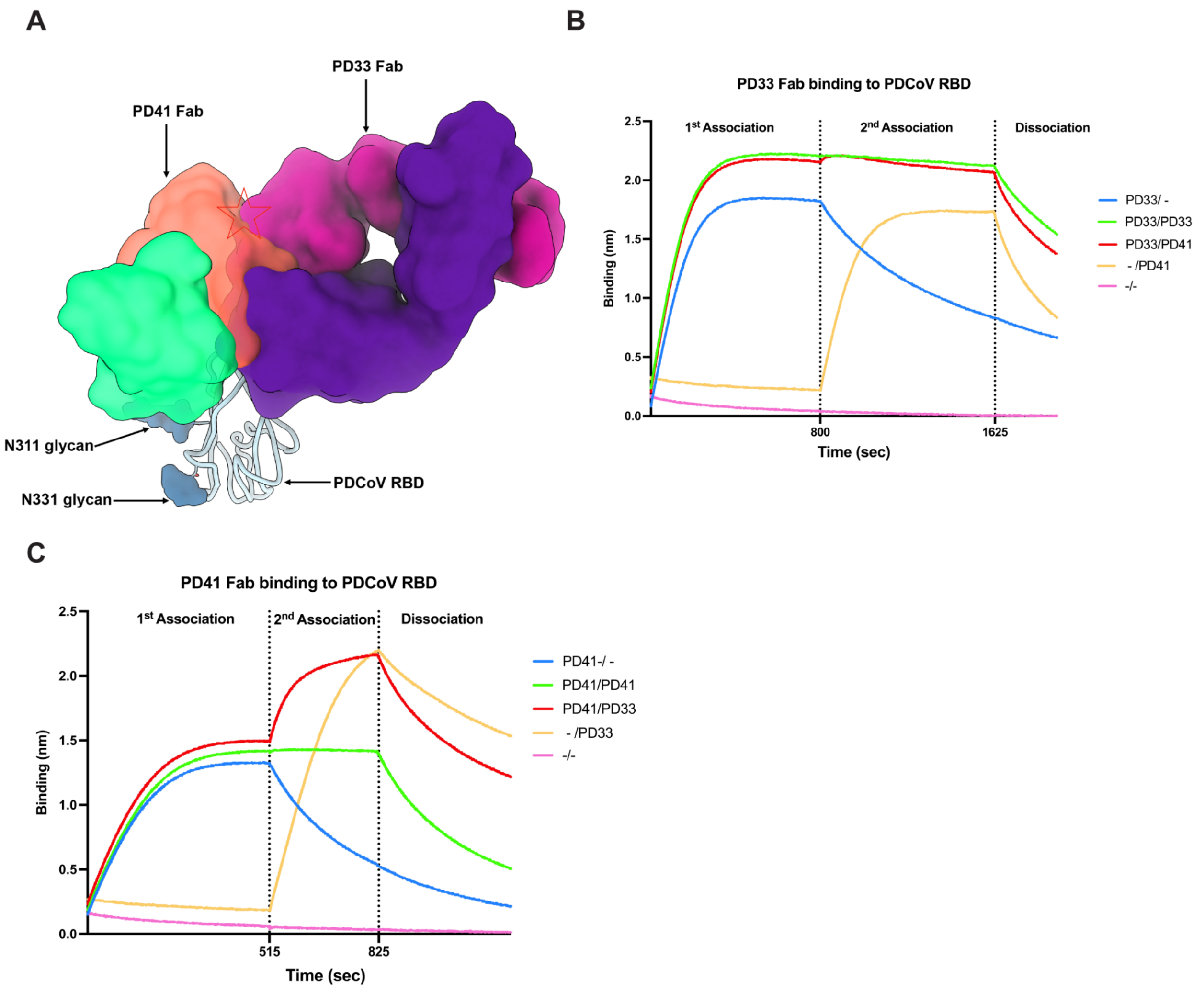

Figure S7: Superimposition of the PD33-bound PDCoV RBD and of the PD41-bound PDCoV RBD$_{SD2018}$ structures showing recognition of distinct but overlapping epitopes. A, PD33 (purple and magenta surfaces for the heavy and light chains, respectively) and PD41 (green and orange surfaces for the heavy and light chains, respectively) recognize overlapping epitopes on the PDCoV RBD (cyan ribbon). N-linked glycans are rendered as blue surfaces. The red star indicates steric clashes. **B,** BLI analysis of Fab PD33 binding to the PDCoV RBD immobilized on biosensors in the presence and absence of Fab PD41. **C,** BLI analysis of Fab PD41 binding to the PDCoV RBD immobilized on biosensors in the presence and absence of Fab PD33.

			K_D (M)	K_D Error	K_a (1/Ms)	K_a Error	K_{dis} (1/s)	K_{dis} Error
PDCoV RBD USA_IL121_2014	PD33	n=1	5.11E-09	4.57E-11	1.15E+05	9.45E+02	5.86E-04	2.05E-06
		n=2	4.36E-09	3.83E-11	1.24E+05	9.80E+02	5.42E-04	2.11E-06
	PD41	n=1	3.72E-08	6.94E-10	5.38E+04	1.00E+03	2.00E-03	2.49E-06
		n=2	2.26E-08	2.60E-10	7.84E+04	8.98E+02	1.77E-03	2.18E-06

bioRxiv preprint doi: https://doi.org/10.1101/2024.03.27.586411; this version posted April 1, 2024. The copyright holder for this preprint (which was not certified by peer review) is the author/funder, who has granted bioRxiv a license to display the preprint in perpetuity. It is made available under aCC-BY 4.0 International license.

Table S1. Table of the KDs, association (Ka) and dissociation rate constants (Kdis) for duplicate lots of PDCoV RBD_{IL121_2014} and Fab fragments of PD33 and PD41.

A

Recombinant DNA	Plasmid	Source	NCBI/Genebank ID
PDCoV RBD USA_IL121_2014	pcDNA3.1 (+)	This study	KJ481931.1
PDCoV RBD HKU15 SD2018_300			MN200481.1
PDCoV RBD HKD_JPN_2016			LC260045.1
PDCoV RBD 110_1197_TW			MZ712033.1
PDCoV RBD Thai_S5011_2015			KU051641.1
PDCoV Spike Full length USA_IL121_2014			KJ481931.1
PDCoV Spike Full length with RBD mutations from SD2018_300			MN200481.1
PDCoV Spike Full length with RBD mutations from HKD_JPN_2016			LC260045.1
PDCoV Spike Full length with RBD mutations from 110_1197_TW			MZ712033.1
PDCoV Spike Full length with RBD mutations from Thai_S5011_2015			KU051641.1
PDCoV Spike Haiti_2022 with two consenus mutations amongst three strains P38L V550A			MW685623
PDCoV Spike Ectodomain IL121_2014 Foldon			KJ481931.1
PDCoV Spike Ectodomain SD2018_300 Foldon			MN200481.1
galline APN full length			ACZ95799.1
galline APN ecto			ACZ95799.1
human APN full length			NP_001141.2
human APN ecto			NP_001141.2

B

Recombinant DNA	RBD Mutations Relative to IL121_2014
PDCoV RBD USA_IL121_2014	N/A
PDCoV RBD HKU15 SD2018_300	M354I, I391V, N397K
PDCoV RBD HKD_JPN_2016	V326I, R342K
PDCoV RBD 110_1197_TW	V326I, T338S
PDCoV RBD Thai_S5011_2015	M349L, T351R

bioRxiv preprint doi: https://doi.org/10.1101/2024.03.27.586411; this version posted April 1, 2024. The copyright holder for this preprint (which was not certified by peer review) is the author/funder, who has granted bioRxiv a license to display the preprint in perpetuity. It is made available under aCC-BY 4.0 International license.

Table S2. Table of the sequence information of PDCoV S variants as full length, ectodomain and RBD along with hAPN and gAPN sequences as full length and ectodomain constructs. A, List of PDCoV variants used in the study and the genebank ID associated for those variants. The table includes information for the APN constructs used in the study and the genebank ID corresponding to those constructs. **B,** List of the PDCoV variants tested in the study and the RBD mutations found in these variants relative to $PDCoV_{IL121\_2014}$.

	PDCoV RBD with PD33	PDCoV Spike SD2018/300 Apo	PDCoV Spike SD2018/300 with one PD41 bound	PDCoV Spike SD2018/300 with PD41 (local refinement)	PDCoV Spike SD2018/300 with two PD41 bound	PDCoV Spike SD2018/300 with three PD41 bound
**Data collection and processing**						
Magnification	105,000	105,000	105,000	105,000	105,000	105,000
Voltage (kV)	300	300	300	300	300	300
Electron exposure (e^{-}/Å^2)	58	44.5	44.5	44.5	44.5	44.5
Defocus range (μm)	0.2 -3.0	0.8 -1.8	0.8 -1.8	0.8 -1.8	0.8 -1.8	0.8 -1.8
Pixel size (Å)	0.835	0.835	0.835	0.835	0.835	0.835
Symmetry imposed	C1	C3	C1	C1	C1	C1
Final particle images (no.)	564,616	24,642	465,175	465,175	99,272	26,452
Map resolution (Å)	3.0	3.1	2.72	3.0	3.0	3.4
FSC threshold	0.143	0.143	0.143	0.143	0.143	0.143
Map sharpening *B* factor (Å^2)	-139	-55.3	-85	- 87.8	-65.8	-38.8
**Validation**						
MolProbity score	1.73			0.87		

bioRxiv preprint doi: https://doi.org/10.1101/2024.03.27.586411; this version posted April 1, 2024. The copyright holder for this preprint (which was not certified by peer review) is the author/funder, who has granted bioRxiv a license to display the preprint in perpetuity. It is made available under aCC-BY 4.0 International license.

Clashscore	3.17			0.2		
Poor rotamers (%)	3.41			0		
Ramachandran plot						
Favored (%)	96.60			95.73		
Allowed (%)	99.23			99.39		
Disallowed (%)	0.77			0.61		

Table S3. CryoEM data collection and refinement statistics.
